# Genetic Creutzfeldt‒Jakob disease with 5-octapeptide repeats presented as frontotemporal dementia

**DOI:** 10.1038/s41439-023-00237-w

**Published:** 2023-03-29

**Authors:** Shinsuke Hamada, Ikuko Takahashi-Iwata, Katsuya Satoh, Tetsuyuki Kitamoto, Hidehiro Mizusawa, Fumio Moriwaka, Ichiro Yabe

**Affiliations:** 1 Department of Neurology, Faculty of Medicine and Graduate School of Medicine, Hokkaido University, Sapporo, Japan; 2 Hokkaido Neurology Hospital, Sapporo, Japan; 3 Department of Health Sciences, Nagasaki University Graduate School of Biomedical Sciences, Nagasaki, Japan; 4 Department of Neurological Science, Tohoku University School of Medicine, Sendai, Japan; 5 National Center of Neurology and Psychiatry, Kodaira, Tokyo, Japan

**Keywords:** Gene amplification, Prion diseases

## Abstract

The N-terminus of the PRNP gene normally contains a 5-octapeptide repeat (R1-R2-R2-R3-R4), and insertions at this locus can cause hereditary prion diseases. In the present study, we found a 5-octapeptide repeat insertion (5-OPRI) in a sibling case of frontotemporal dementia. Consistent with previous literature, 5-OPRI rarely met the diagnostic criteria for Creutzfeldt‒Jakob disease (CJD). We propose 5-OPRI as a suspected causative mutation for early-onset dementia, especially the frontotemporal type.

Creutzfeldt‒Jakob disease (CJD) is a prion protein-mediated zoonosis that typically presents with subacute progressive dementia, leading to akinesia and mutism. Since abnormal prion proteins are more resistant to sterilization by heat and protein denaturation than viruses and bacteria, the prevention of prion infection is of the utmost importance. In Japan, prion disease surveillance began in 1999 to identify all patients with prion diseases, and prion-related protein analyses using cerebrospinal fluid (CSF) and PRNP gene analysis have continued as important diagnostic tests. The N-terminus of the PRNP gene normally contains an octapeptide repeat (R1-R2-R2-R3-R4). Insertions in this repeat have been reported to cause hereditary prion diseases in a small number of cases^1^. This mutation is rarer than the causative mutations for some other hereditary prion diseases, and its clinical traits depend on the number of repeat insertions; however, the details remain unclear, especially in cases with 5-octapeptide repeat insertion (5-OPRI)^2^. Recently, we found a case of siblings with 5-OPRI in which diagnosis was possible only by genetic analysis. We propose 5-OPRI as a suspected causative genetic mutation for early-onset dementia, especially the behavioral variant of frontotemporal dementia.

Patient 1 (Fig. 1a: III–1) had been aware of memory loss and calculation difficulties since the age of 56. His work colleagues mentioned that his behavior was impulsive, compulsive, and socially inappropriate (e.g., deviating from typical manners). He was diagnosed with Parkinsonism at the age of 58 years by a family physician and visited a neurology hospital. The patient’s father (Patient 3, Fig. 1a: II–1) was diagnosed with Pick’s disease, and the paternal grandmother (Fig. 1: I–2) was diagnosed with dementia at the age of 50 years and died at 61. Patient 1 showed conspicuously strange behavior and cognitive decline (Mini-Mental State Examination (MMSE) 22/30, Frontal Assessment Battery (FAB) 13/18, Raven’s Colored Progressive Matrices (RCPM) 25/37), a positive Myerson sign, cogwheel muscle rigidity in both upper limbs, and an unstable gait. There was no myoclonus detected. Blood tests revealed no abnormalities, and CSF tests revealed no abnormalities in cell count, total protein, total tau protein, 14-3-3 protein, or real-time quaking-induced conversion (RT-QUIC). Electroencephalography (EEG) revealed an 8–11 Hz fluctuation in the basic rhythm but no paroxysm, including periodic synchronous discharge (PSD). Diffusion-weighted imaging (DWI) revealed mild cortical atrophy in the frontotemporal lobe and enlargement of the lateral and third ventricles without cortical hyperintensity (Fig. 1b, c).

**Fig. 1:**
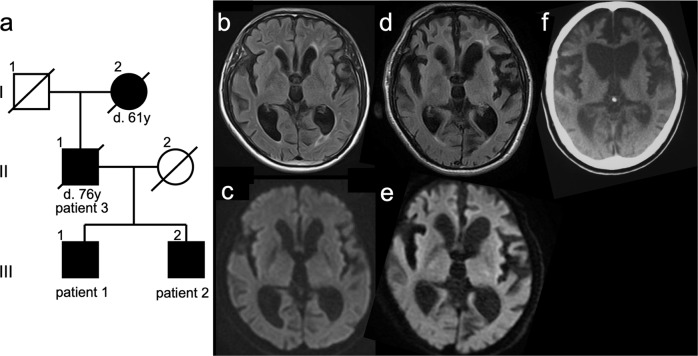
Family pedigree and brain imaging findings for patients. **a** Family pedigree. **b** Fluid-attenuated inversion recovery (FLAIR) magnetic resonance imaging (MRI) of patient 1 at 1.5 tesla. **c** Diffusion-weighted imaging (DWI) of patient 1. **d** FLAIR imaging of patient 2. **e** DWI of patient 2. **f** Computed tomography (CT) imaging of patient 3.

Patient 2 (Fig. 1: III–2) was the younger brother of patient 1. He had had frequent falls since the age of 41 years, and his family doctor diagnosed dementia with disorientation at the age of 42. He showed parkinsonian symptoms at the age of 47 years, and from the age of 48, he developed delirium and agitation at night, as well as socially inappropriate and compulsive behavior, behavioral inertia, and diminishing social interest. At 51 years of age, he could not walk, and at 54, he became bedridden. He was admitted to the neurology hospital at the age of 54 years for a thorough examination. He had difficulty understanding instructions due to severe cognitive impairment and showed a positive Myerson sign and palmar-mandibular reflex, tonicity in both upper limbs, hyperreflexia of tendons in both lower limbs, and positive abnormal reflexes. Blood test results were normal, and CSF examination revealed a normal cell count, elevated total protein (90 mg/dl), normal total tau protein, 14-3-3 protein, and a negative finding on RT-QUIC. EEG showed a basic rhythm of 8–11 Hz without paroxysms, including PSD. DWI revealed generalized atrophy of the cerebral cortex and ventricular enlargement without cortical hyperintensity (Fig. 1d, e).

Patient 3 was the father of patients 1 and 2. He presented with impulsive behavior, personality changes that had diminished his personal warmth, frequent falls, and repetitive movements at the age of 49; years, dysarthria at age 52; and gait disturbance and dementia at age 53. At the initial visit to a neurologist at the age of 56 years, he had severe dementia, frontal release signs including palmomental reflex and grasping, repetitive movements, dysarthria, dressing apraxia, hyperreflexia of both upper limb tendons, and impairment of the postural reflexes. EEG revealed a basic rhythm of 9–11 Hz with no paroxysms, including PSD. Brain computed tomography revealed generalized atrophy of the frontotemporal cerebral cortex and ventricular enlargement (Fig. 1f).

Prion protein gene testing was performed in patients 1 and 2,1, and genetic CJD (gCJD) was diagnosed based on the presence of five identical insertion mutations in the OPR region in both cases: (R1-R2-R2-R3g-R2-R3g-R2-R2-R3-R4), (NC_000020.11:g.4699465_4699466ins[GCAGCCTCATGGTGGTGGCTGGGGGCAGCCCCAT;4699380_4699465]). Patient 1 was found to be heterozygous for methionine-valine at codon 129 and homozygous for glutamine at codon 219, and the 5-OPRI mutation was found on the M allele side. Patient 2 was found to be homozygous for methionine at codon 129 and glutamine at codon 219.

Our literature search found 20 patients with reported 5-OPRI mutations across nine families in six research articles, including this report (Table 1)^1,3–7^. Pathological diagnosis was performed on 10 patients. Only two families had common mutations (KE and NI). The mean age at onset was 45 years (standard error, 2.53), and only four patients had a total disease duration of <2 years. Progressive dementia was present in all cases, and descriptions of findings suspicious for frontotemporal dementia (e.g., behavioral disinhibition; apathy or behavioral inertia; loss of sympathy or empathy; perseverative, stereotyped or compulsive/ritualistic behavior; hyperorality and dietary changes; and frontal release signs) were found in 14 patients. MRI was performed in 12 patients, but DWI and fluid-attenuated inversion recovery (FLAIR) imaging, which are typically helpful in diagnosing CJD, were described in only four patients, and none of them showed abnormal signals. Electroencephalography was performed in 10 patients, and PSD was noted in only two. The 14-3-3 protein levels were measured in five patients, one of whom tested positive.

**Table 1:** Characteristics, clinical symptoms, and laboratory findings of patients with confirmed 5-octapeptide repeat insertion mutations.

Patient (references)	Mutation	Pathology	Codon 129	Age at onset	Disease duration (months)	Dementia	Clinical findings supporting behavioral variant frontotemporal dementia	Myoclonus	Ataxia	Pyramidal	Extra pyramidal	Mutism	EEG^a^	MRI or other brain imaging		CSF^c^	
													PSD^b^	Hyperintensity in DWI/FLAIR	Atrophy site on brain imaging	14-3-3	RT-QUIC^d^
KE1(1)	122323g2234	+		31	174	+	Socially inappropriate behavior	+	+		+						
KE2(1)	122323g2234	+		45	60	+	Inertia, diminished social interest		+	+							
UK1(3)	1223222234	+	MM	42	87	+	Socially inappropriate behavior, apathy	+		+	+	+	−		Diffuse	−	
UK2(3)	1223222234		MM	44	>84	+	Caress actions, apathy, compulsive behaviors, stereotypy of speech	+					−				
UK3(3)	1223222234		MM	26	>144	+			+	+	+						
GE1(4)	1223g3g3g2234	+		51	27	+			+				−		Left frontal lobe		
GE2(4)	1223g3g3g2234	+	MV	61	96	+	Apathy, inertia, diminished social interest		+	+							
GE3(4)	1223g3g3g2234	+	MV	52	4	+		+	+	+			+		Diffuse		
JA1(5)	122a223g2234		MM	45	>48	+		−	+	+					Cerebral cortex, vermis		
SA1(6)	1232323g234	+		51	18	+	Socially inappropriate behavior	+	+			+	−		Diffuse		
SA2(6)	1232323g234			40	84	+	Socially inappropriate behavior	+	+			+					
SA3(6)	1232323g234				<60	+											
SA4(6)	1232323g234				<60	+											
EN1(6)	1223g222234	+		39	48	+	Inertia, diminished social interest		+								
EN2(6)	1223g222234	+	MV	61	168	+	Socially inappropriate behavior, inertia, diminished response to other people’s needs and feelings	−	+						Cerebral cortex		
EN3(6)	1223g222234		MM	34	>120	+	Impulsive, rash actions, oral exploration, frontal release signs		+				−		Bilateral parietal lobes		
NI1(6)	122323g2234		MM	63	10	+	Impulsive, rash actions	+	+	+		+	+	−	Cerebral cortex	+	
DU1(7)	1222222234	+	MM	35	92	+	Apathy, diminished social interest, stereotypy of speech	−	−	−	+	−		−	diffuse	−	
HO1	1223g23g2234		MV	56	>120	+	Socially inappropriate behaviour, loss of manners, rash actions, apathy, compulsive behaviours	−	−	−	+	−	−	−	Diffuse	−	−
HO2	1223g23g2234		MM	41	192	+	Socially inappropriate behaviour, Inertia, dimished social interest, compulsive behaviours	−	−	+	+	−	−	−	Frontotemporal lobes	−	−

HO1 and HO2: Patient 1 and 2 in our presented cases.

^a^Electroencephalography.

^b^Periodic synchronous discharge.

^c^Celebrospinal fluid.

^d^Real-time quaking-induced conversion.

The two reported cases showed no cortical hyperintensities on DWI/FLAIR images, no PSD on EEG, and negative 14-3-3 protein and RT-QUIC. According to our literature review, gCJD caused by 5-OPRI mutations has a markedly younger onset and a longer disease duration than sCJD. Many patients lack myoclonus and exhibit symptoms suggesting the behavioral variant of frontotemporal dementia^8^. There is no typical PSD on EEG, and there are no cortical hyperintensities on DWI or FLAIR brain MRI, although this has been confirmed only in recent reports. Since the 5-OPRI mutation is capable of infecting other organisms, as revealed in experimental infection studies, the recommendation is to pursue a genetic diagnosis of gCJD aggressively, even if the case is not typical of sCJD.

Patients with gCJD due to 5-OPRI mutations show the behavioral variant of frontotemporal dementia from a young age, do not present with myoclonus, and have no obvious abnormalities in either EEG or cortical signals on MRI. These diseases do not meet the usual diagnostic criteria for prion diseases, and the utility of genetic diagnosis must be emphasized.
